# CT volumetric measurement of colorectal cancer helps predict tumor staging and prognosis

**DOI:** 10.1371/journal.pone.0178522

**Published:** 2017-06-01

**Authors:** Jin Young Park, Se Hyung Kim, Sang Min Lee, Jeong Sub Lee, Joon Koo Han

**Affiliations:** 1 Dongnam Institute of Radiological and Medical Sciences Cancer Center, Busan, Korea; 2 Department of Radiology, Seoul National University Hospital, Seoul, Korea; 3 Department of Radiology, Seoul National University College of Medicine, Seoul, Korea; 4 Department of Radiology, Hallym University Sacred Heart Hospital, Anyang, Korea; 5 Department of Radiology, Jeju National University Hospital, Jeju, Korea; 6 Institute of Radiation Medicine, Seoul National University Medical Research Center, Seoul, Korea; Chang Gung Memorial Hospital Kaohsiung Branch, TAIWAN

## Abstract

**Purpose:**

To evaluate feasibility of CT colonography (CTC) volumetry of colorectal cancer (CRC) and its correlation with disease stage and patients’ survival.

**Materials and methods:**

CTC volumetry was performed for 126 patients who underwent preoperative CTC. Reproducibility of tumor volume (Tvol) between two readers was assessed. One-way ANOVA and ROC analysis evaluated correlation between Tvol and pTNM staging. ROC analysis compared diagnostic performance to predict pTNM staging between Tvol and radiologist. Kaplan-Meier test compared overall survival.

**Results:**

Reproducibility among readers was excellent (interclass correlation = 0.9829). Mean Tvol showed an incremental trend with T stage and Tvol of pT4b stage was significantly larger than other stages (P<0.0001). Az value (0.780) of Tvol to predict pT4b stage was significantly larger than that (0.591) of radiologist (P = 0.004). However, Tvol was not significantly different according to pN stage. Az values (0.723~0.857) of Tvol to predict M1 or M1b were comparable to those (0.772~0.690) of radiologist (P>0.05). Smaller tumor burden (≤12.85cm^3^), ≤T3, N0, M0 stages, and curative surgery were significantly associated with patients’ longer survival (P<0.05).

**Conclusion:**

CT volumetry has a limited value to predict N stage; however, it may outperform the radiologist’s performance when predicting pT4b and M1b stage and can be a useful prognostic marker.

## Introduction

Colorectal cancer (CRC) is the third most common cancer and the third leading cause of cancer death in the United States with an estimated 137,000 cases diagnosed annually [1]. The prognosis of CRC is closely related to stage at diagnosis; therefore, the preoperative staging system should help differentiate tumors with a good prognosis from those with poor prognosis to facilitate an appropriate therapy protocol and optimize outcomes. CRC staging incorporates the depth of wall invasion (T stage), the presence of lymph node (N stage), and distant metastases (M stage). CT is most commonly used for CRC staging among the several radiologic staging modalities, such as endoscopic ultrasonography (EUS), computed tomography (CT), magnetic resonance imaging (MRI), and position emission tomography (PET). CT colonography (CTC) is notably used as a preoperative diagnostic and staging tool for CRC due to the promising results it provides in the diagnosis of colorectal polyps and cancers. However, the reported performance of CT for T and N staging of CRC is variable (sensitivity and specificity for tumor invasion, 55%-100% and 42%-94%; sensitivity and specificity for nodal metastasis, 13%-92% and 55%-98%, respectively) [2, 3]. Improved accuracy has been reported using CT with three-dimensional reconstructed images [4–8]; however, there is opportunity to further improve the locoregional staging of CRC.

CT volumetric parameter has gained acceptance as a prognostic marker for gastric cancer [9] since Kikuchi et al. reported the usefulness of CT volumetry to predict patients’ survival as well as predict TNM staging in gastric cancer [10, 11]. In terms of rectal cancer, there have been several reports showing the usefulness of CT or MR volumetry to predict treatment response after chemoradiation therapy [12–15]. However, to the best of our knowledge, there have been no reports that investigate the feasibility and usefulness of CTC volumetric evaluation to predict TNM staging and patients’ prognosis. Therefore, this study evaluates the feasibility of performing CTC volumetry of CRC and its correlation with disease stage and patients’ survival.

## Materials and methods

This retrospective study was approved by the institutional review board of our institute (Seoul National University Hospital) and informed consent was waived.

### Patients

We obtained a list of 363 patients who underwent CTC between November 2008 and December 2010 from our radiology database. Only patients who fulfilled the following criteria were included: (a) patients surgically proven to have CRCs, (b) patients who had preoperative contrast-enhanced CTC images with optimal colonic distention for volumetric assessment of primary CRCs, (c) patients who did not receive any treatment before CTC. From the above criteria, we excluded 237 patients who underwent only polypectomy for colorectal polyps (n = 196), treated with neoadjuvant chemotherapy or radiation therapy (n = 11), had deployed colorectal stent for obstructing CRCs (n = 12), proven to have histopathologic results other than adenocarcinoma, such as neuroendocrine tumor or lymphoma (n = 2), had CTC images with suboptimal colonic distention (n = 2), or underwent only precontrast CTC examination (n = 14). Finally, 126 patients (71 men and 55 women; mean age, 63.7 years; range, 32–86 years) with surgically proven CRCs comprised our study population.

### Clinical and histologic features

Clinical features were analyzed by one author (J.Y.P.) using electronic medical records (EMR) from our hospital. She reviewed clinical features, pathologic TNM staging, histologic type, type of surgery, and presence and type of adjuvant chemotherapy. All resected CRC were staged according to American Joint Committee on Cancer (AJCC) guidelines 7^th^ edition and histological type was categorized based on World Health Organization (WHO) classifications. We also collected clinical outcomes that included patients’ current status (dead, alive, or follow-up loss) and survival days after the initial diagnosis of CRCs. Overall follow-up survival data was completed by reviewing the EMR of our hospital as well as by contacting the Resident Service Division of the Ministry of Public Administration and Security. Patients were censored if they were dead or follow-up loss at the time point of November 20, 2015. Table 1 summarizes the clinical and histologic features of 126 patients with CRCs.

**Table 1:** Clinical and histological features of 126 patients with colorectal cancer.

		Patients
M:F		71:55
Mean age (range)		63.7 years (32–86 years)
Histology	WD tubular adenocarcinoma	6
	MD tubular adenocarcinoma	111
	PD tubular adenocarcinoma	7
	Mucinous adenocarcinoma	2
Pathologic T stage (pT)	T1	6
	T2	5
	T3	91
	T4a	14
	T4b	10
Pathologic N stage (pN)	N0	52
	N1	35
	N2	6
Pathologic M stage (pM)	M0	93
	M1a	27
	M1b	6
Type of surgery	Right hemicolectomy	11
	Left hemicolectomy	10
	Anterior resection	60
	Low anterior resection	40
	Hartmann’s operation	1
	Subtotal colectom	1
	Endoscopic submucosal dissection	1
	Colectomy with adjacent organ resection	2
Intent of surgery	Curative (R0 resection)	96
	Palliative (R1 or R2 resection)	30

WD = well-differentiated, MD = moderately differentiated, PD = poorly differentiated, T = tumor, N = node, M = metastasis

### CT colonography techniques

Bowel preparation for CTC started two days before examination. All patients were asked to refrain from foods rich in fiber, seeded fruits, and seaweed. Patients had a regular diet for breakfast and rice porridge for lunch. No dinner was allowed. For fecal tagging, we used 50 ml of water-soluble iodinated contrast agent (Gastrografin^®^, Bayer-Schering, Berlin, Germany) as a tagging regimen. Gastrografin was consumed at 9:00 PM, 1 day before the CTC examination. At 6:00 PM of the same day, physical cleansing of the colon was started with 250 ml of orally administered magnesium citrate solution (Magcorol Soln^®^, Taejoon Pharm Co., Seoul, Korea) followed by the ingestion of four tablets of bisacodyl (total 20 mg) at 8:00 PM. Patients were requested to drink a glass of water every 1 hour from 6:00 to 9:00 PM. No food was consumed on the morning of the procedure. Ten minutes before CTC examination, all patients received a slow intravenous bolus of a hypotonic agent (20 mg of Buscopan^®^, Boehringer Ingelheim Korea, Seoul, Korea) unless contraindicated to reduce peristalsis and to minimize abdominal discomfort.

A dedicated CT technician gently inserted a flexible small rectal tube with a retention cuff into patients in a left lateral decubitus position and carefully insufflated the colon using an automated CO_2_ delivery system (PROTOCO2L, E-Z-EM, Westbury, NY, USA) to a maximum rectal pressure of 25 mmHg. To ensure sufficient colon distension, a CT digital radiograph was acquired and further insufflation was performed when colonic distension was insufficient or collapsed colonic segments were identified. Images were acquired first in supine and then prone positions for every patient. In the prone position, a specially designed cushion was placed under the patient’s abdomen so as not to compress the abdomen on the CT table. A prone digital radiograph was acquired and additional insufflation was performed if the colonic distension seemed suboptimal. CT was performed with 64-row MDCT (Brilliance 64, Philips Medical Systems, Cleveland, OH, USA). For supine position, precontrast scanning was performed while contrast-enhanced CT scanning 60 seconds after contrast administration was done for the prone position. An iodinated contrast agent of 1.5 mg/kg (Ultravist 370, Bayer Schering Pharma, Berlin, Germany) was used at a rate of 3–5 ml/sec. Imaging parameters for CTC were: detector configuration, 64x0.625 mm; pitch, 1.172; gantry rotation time, 0.72 seconds; slice thickness, 1 mm; reconstruction increment, 0.7 mm; matrix, 512 ×512, and a field of view to fit. For kVp and mAs settings, 120 kVp and 50 effective mAs was used in supine position and 120 kVp and 200 mAs in prone position.

### CT volumetry

**Fig 1:**
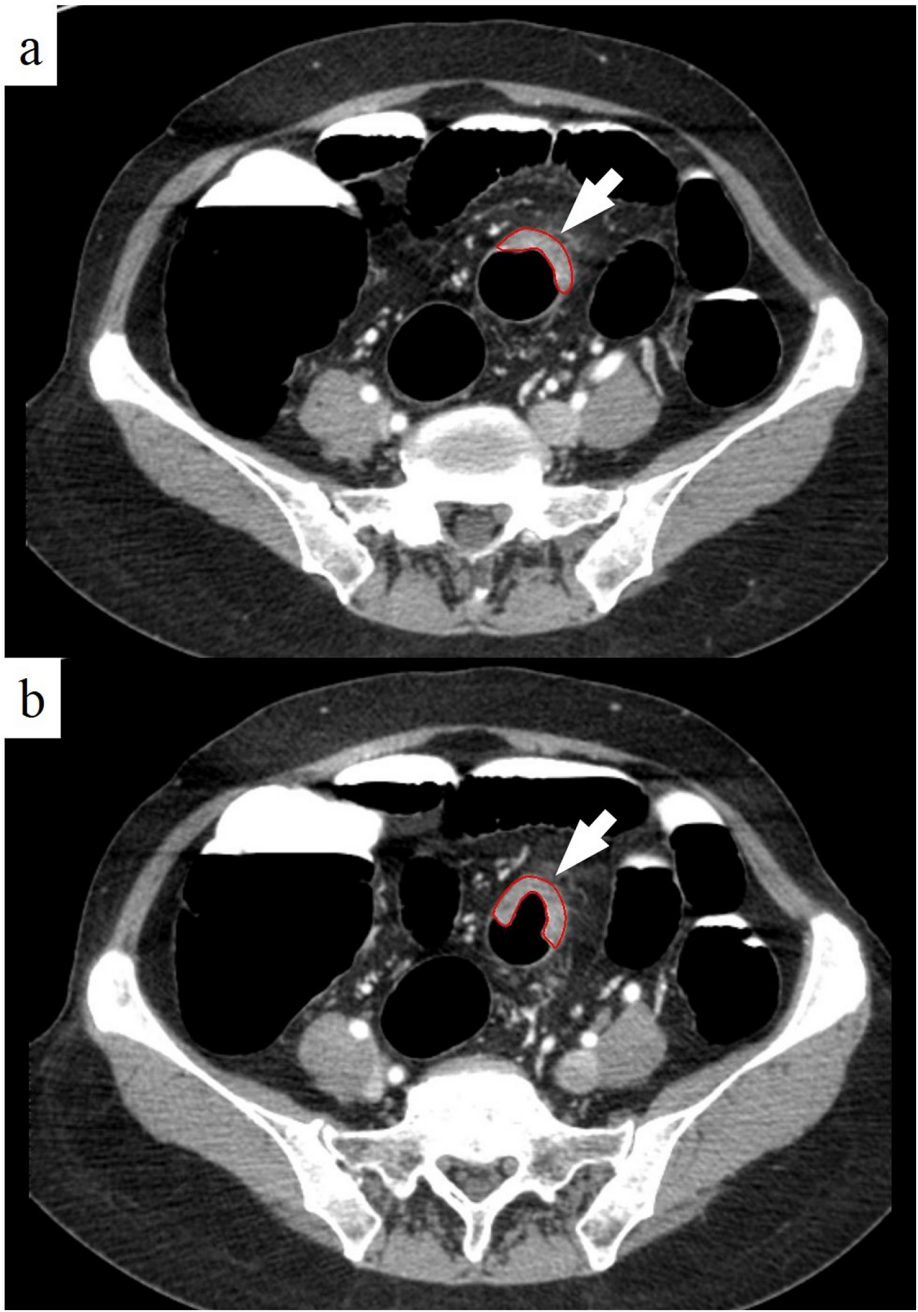
Volume measurement using CT colonography. **a, b.** Eccentric enhancing wall thickening (arrow) of the sigmoid colon is well demonstrated on axial CT images in prone position. Regions of interest (ROIs) (red line) are drawn covering abnormal wall thickening on every contiguous CT slices. Volume (cm^3^) is calculated by multiplying areas (cm^2^) and reconstruction interval (cm).

Volumetric measurement for CRCs was independently performed by two radiologists (J.Y.P. and J.S.L. with 5 years of experience for abdominal imaging) blinded to surgical or histopathologic results using an area measurement function installed in our picture archiving and communication system (PACS; M-view, INFINITT, Seoul, Korea). Contrast-enhanced CTC images obtained on prone position were primarily used for volume measurement. We also refer to non-contrast CTC images obtained on supine position to accurately delineate the margin of the cancers. Two-dimensional (2D) areas of the tumor, defined as enhancing wall thickening that showed different attenuation and contour from normal adjacent colorectal wall, were measured in all 2D images in which tumors were included by manually tracing the lesion boundary. Pericolic lymph nodes, vessels and adjacent viscera were carefully excluded. The volumes of the lesions were calculated by adding each of the 2D volumes (multiplying 2D area by reconstruction interval) of the entire lesion (Fig 1). The time required to manually trace the lesion boundary using region-of-interest (ROI) function and processing the data for volume calculation was recorded.

### Conventional CT staging by radiologist

A different radiologist (S.M.L. with 8 years of experience for abdominal imaging) blinded to surgical or histopathologic results evaluated the TNM staging of CRCs based on American Joint Committee on Cancer (AJCC) 7^th^ edition guidelines. The radiologist was asked to determine TNM staging using a 5-point confidence scale. For T staging, a confidence level to predict T1 vs ≥ T2, ≤ T2 vs ≥ T3, ≤ T3 vs ≥ T4a, and ≤ T4a vs T4b was recorded. As an example, to differentiate T1 from ≥ T2 stage, 1 was defined as definitely T1 stage, 2 as probably T1, 3 as possibly ≥ T2, 4 as probably ≥ T2, and 5 as definitely ≥ T2. For N staging, a confidence level to predict N0 vs N1 was also recorded using a 5-point scale; 1, definitely N0; 2, probably N0; 3, possibly ≥ N1; 4, probably ≥ N1; and 5, definitely ≥ N1. A confidence level to predict M0 vs M1 and ≤ M1a vs M1b was recorded for M staging.

### Statistical analysis

Interobserver agreement between two radiologists for Tvol measurement was assessed using interclass correlation (ICC). One-way analysis of variance (ANOVA) test or independent t-test was used to analyze significant differences between Tvols of different T stages, N stages, and M stages. A receiver operating characteristics (ROC) analysis was used to predict TNM stages by Tvol. For comparison of performance between Tvol and radiologist in predicting TNM stages, an ROC analysis with DeLong method was used to test the statistical significance of the difference between the areas under different ROC curves.

A Kaplan-Meier method and log-rank test analyzed the survival curves of all 126 patients; in addition, the effect of patient age (< 70 years or ≥ 70 years), sex, type of surgery (curative vs palliative), pTNM stage (≤ T3 vs T4, N0 vs ≥ N1, and M0 vs M1) and tumor volume (< 12.85 cm^3^ or ≥ 12.85 cm^3^) on patients’ survival were also analyzed. SPSS 22.0 for windows (SPSS Inc., Chicago, IL, USA) and MedCalc version 15.11 (MedCalc software, Mariakerke, Belgium) performed statistical analysis. P<0.05 was considered to indicate statistical significance.

## Results

### Feasibility and reproducibility

CT volumetry was successfully performed on all patients by two radiologists. Interobserver agreement between the two readers was excellent (interclass correlation = 0.9829; 95% CI, 0.9757 to 0.9879). Therefore, final Tvol was used by averaging the results of the two radiologists. Mean measurement time for each patient by radiologists 1 and 2 was 6.22 minutes (range, 0.80–21.02 minutes) and 4.92 minutes (range, 0.38–16.38 minutes), respectively.

### Surgery and pathologic results

Pathologic TNM (pTNM) staging was available in all patients due to the inclusion of patients who only had surgically confirmed CRCs. Among a total of 126 patients, 11 patients underwent right hemicolectomy, 10 left hemicolectomy, 60 anterior resection (AR), 40 low anterior resection (LAR), 1 Hartmann’s operation, and 1 subtotal colectomy. One patient received endoscopic submucosal dissection. En-bloc resection of adjacent organ with colectomy (left hemicolectomy in one and anterior resection in the other) was performed in the remaining two pT4b stage patients. Ninety six patients received curative R0 resection while the remaining 30 patients received palliative R1 (n = 4) or R2 (n = 26) resection. Of 126 patients, 6 had pT1, 5 pT2, 91 pT3, 14 pT4a, and 10 pT4b; 52 N0, 35 N1, and 39 N2; 93 M0, 27 M1a, and 6 M1b. For 33 patients with M1 staging, histopathologic results (n = 22) or imaging results (n = 11) using abdominopelvic CT, chest CT, liver MRI, PET/CT or PET/MRI during > 1 year follow-up were used. For 27 M1a, the most frequent metastatic organ was liver (n = 14), followed by lung (n = 6), peritoneum (n = 4), paraaortic lymph node (n = 2), and adrenal gland (n = 1). Of 6 patients with M1b, liver and lymph node (n = 2) and seeding and lymph node (n = 2) are the most frequent sites, followed by lymph node and lung (n = 1) and liver, lung, lymph node, and seeding (n = 1). Most of cancers (97.6%, 123/126) were tubular adenocarcinomas; 6 well-differentiated, 111 moderately differentiated, and 7 poorly differentiated. The remaining two cancers were mucinous adenocarcinomas.

### Relationship between tumor volume and pTNM staging

The mean Tvol of CRC showed an incremental trend with T stage (T1 = 3.74 cm^3^; T2 = 14.04 cm^3^; T3 = 27.08 cm^3^; T4a = 27.56 cm^3^; and T4b = 71.06 cm^3^) with statistical difference between stages (P<0.0001) (Table 2). In particular, Tvol of pT4b stage was significantly larger than other stages on the post-hoc test (P<0.05). On ROC analysis, the area under the ROC curve (Az) of Tvol to differentiate T1 stage from ≥ T2 stage was 0.958 (P<0.0001). At a cut-off value of 6.53 cm^3^, sensitivity of 95.83% and specificity of 83.33% were achieved. Tvol predicted ≥ T3, ≥ T4a, and T4b tumors with Az values of 0.855 (P<0.0001), 0.667 (P = 0.0058), and 0.780 (P<0.0001) respectively (Table 3). However, the mean Tvol of CRC among N stages was not significantly different (N0 = 29.76 cm^3^; N1 = 29.43 cm^3^; and N2 = 27.57 cm^3^) (P = 0.951) (Table 2). In addition, the Az value of Tvol to differentiate N0 stage from ≥ N1 stage was around 0.5 (0.532) and was not significant (P = 0.542) (Table 3).

**Table 2:** Mean and standard deviations of tumor volumes in each TNM stage.

Stage		T Stage	N Stage	M Stage
0	0		29.76 ± 34.19 cm^3^ (n = 52)	25.77 ± 35.67 cm^3^ (n = 93)
1	1a	3.74 ± 5.03 cm^3^ (n = 6)	29.43 ± 46.79 cm^3^ (n = 35)	33.23 ± 18.98 cm^3^ (n = 27)
	1b			59.87 ± 43.90 cm^3^ (n = 6)
2	2	14.04 ± 9.77 cm^3^ (n = 5)	27.57 ± 15.43 cm^3^ (n = 6)	
3	3	27.08 ± 23.89 cm^3^ (n = 91)		
4	4a	27.56 ± 15.92 cm^3^ (n = 14)		
	4b	71.06 ± 85.02 cm^3^ (n = 10)		
P value*		***<0*.*0001***	0.951	***0*.*042***

TNM = tumor, node, and metastasis.

*P values were obtained using one-way ANOVA test. P values in ***Italic Bold*** indicate a statistical significance.

**Table 3:** Az value, optimal cut-off value, sensitivity, and specificity to differentiate TNM Staging.

Stage	Differentiation	Az Value	Optimal Cut-Off Value	Sensitivity	Specificity	P Value
T Stage	T1 vs ≥T2	0.958	6.53 cm^3^	95.83%	83.33%	***<0*.*0001***
	≤T2 vs ≥T3	0.855	12.85 cm^3^	80.87%	81.82%	***<0*.*0001***
	≤T3 vs ≥T4a	0.667	26.48 cm^3^	58.33%	70.59%	***0*.*0058***
	≤T4a vs T4b	0.780	19.18 cm^3^	100%	50%	***<0*.*0001***
N Stage	N0 vs ≥N1	0.532				0.542
M Stage	M0 vs M1	0.723	27.50 cm^3^	63.64%	76.34%	***<0*.*0001***
	≤M1a vs M1b	0.857	29.37 cm^3^	100%	72.5%	***<0*.*0001***

Az = area under the receiver operating characteristic curve. TNM = tumor, node, and metastasis. P values in ***Italic Bold*** indicate statistical significance.

**Fig 2:**
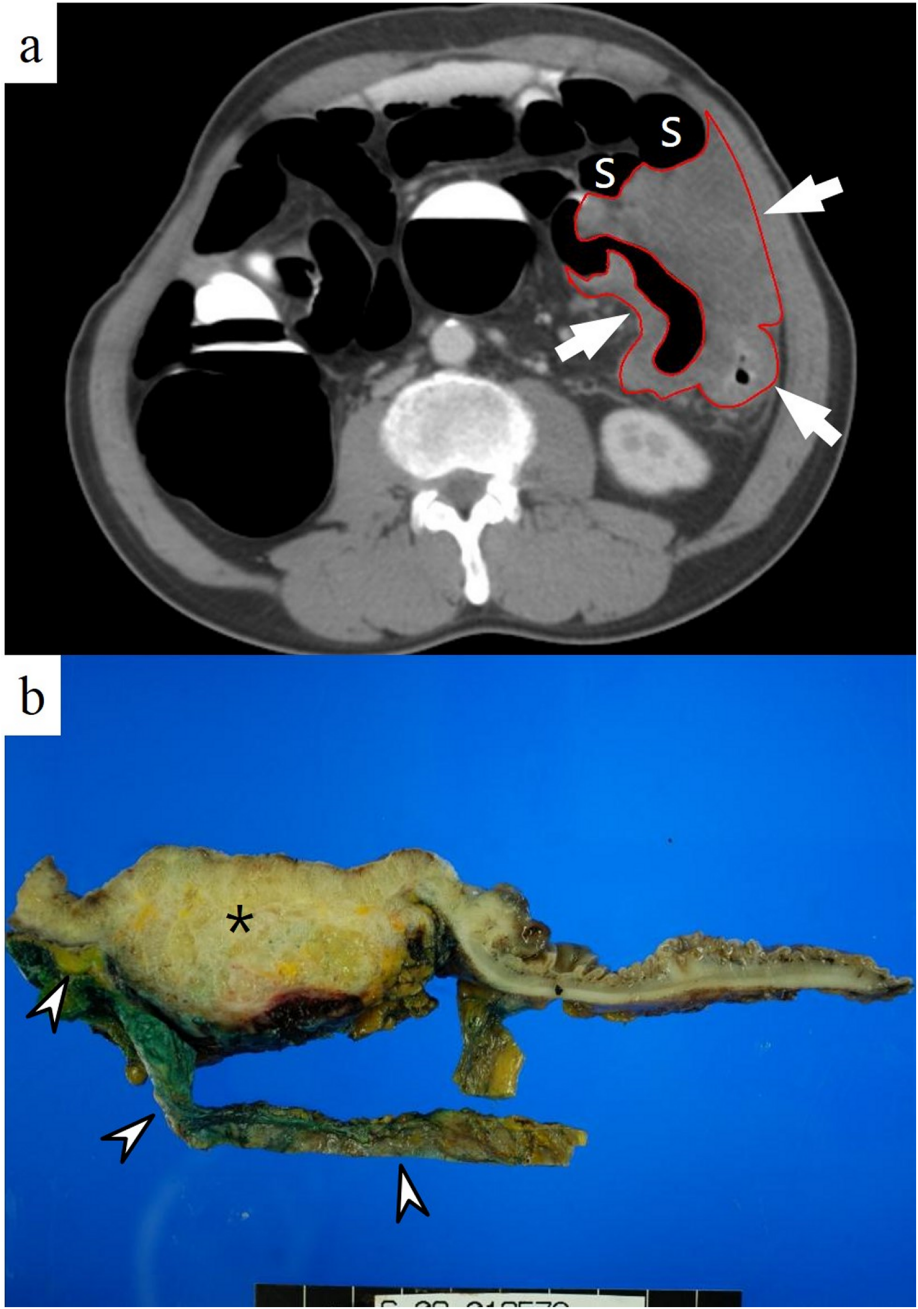
A 56-year old man with a T4b cancer at the descending colon. (**a**) A huge low attenuating mass (arrows, red line) is well depicted on axial CT images in prone position. Tumor volume was 297.20 cm^3^. The mass closely abuts the small bowel loops (S) anteriorly. However, the radiologist considered this lesion as T4a stage with a confidence level of 2 (probably ≤ T4a). On operative field, tumor directly invaded into the ileum; therefore, en bloc resection including descending, sigmoid colons and attached ileum was performed. (**b**) A photograph of gross specimen after left hemicolectomy shows a bulky ulceroinfiltrative mass (*) at the colon with direct invasion to the adjacent ileum (arrowheads). Final histopathology confirmed a mucinous adenocarcinoma with pT4bN1bM0 stage (not shown).

The mean Tvol of CRC showed an incremental trend with M stage (M0 = 25.77 cm^3^; M1a = 33.23 cm^3^; and M1b = 59.87 cm^3^) with statistical difference between stages (P = 0.042) (Table 2). In particular, Tvol of M1b stage was significantly larger than M0 stage on post-hoc test (P = 0.016). On ROC analysis, Az value to differentiate M0 stage from M1 stage was 0.723 (P<0.0001). A cut-off value of 27.50 cm^3^, sensitivity of 63.64% and specificity of 76.34% were achieved. In addition, Tvol predicted M1b tumors with Az, sensitivity, and a specificity of 0.857, 100%, and 72.5% at a cut-off value of 29.37 cm^3^, respectively (P<0.0001) (Table 3). Fig 2 represents an example of T4b stage.

### Comparison between tumor volume and radiologist for staging colorectal cancer

**Fig 3:**
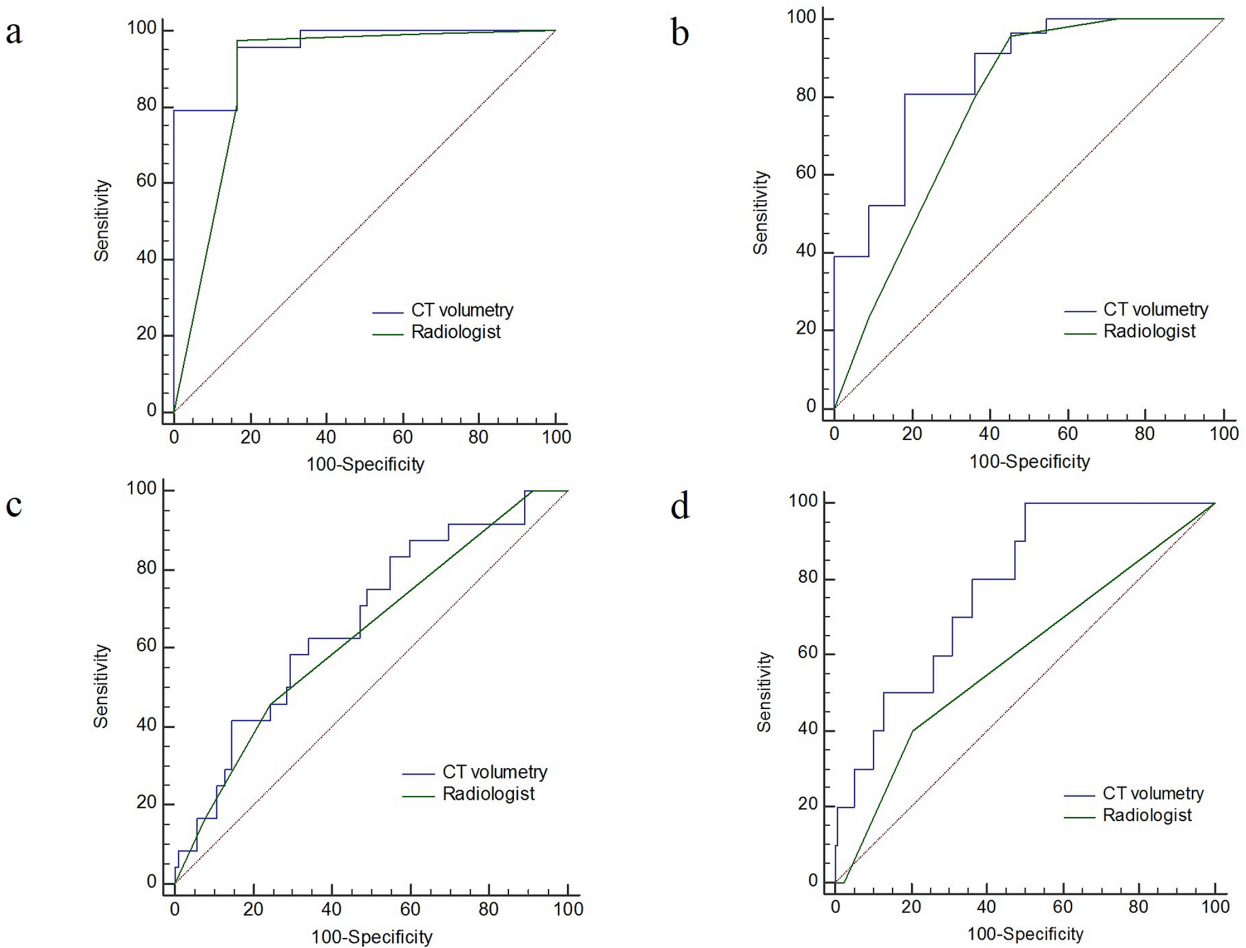
Receiver operating characteristic (ROC) curves of CT volumetry and conventional CT staging by radiologist to predict ≥ T2 (**a**), ≥ T3 (**b**), ≥ T4a (**c**), and T4b (**d**) stage of colorectal cancer. **d.** To predict T4b stage, the area under the ROC curve (Az, 0.780) of CT volumetry is significantly larger than that (0.591) of the radiologist (P = 0.004). For other T stages, Az values of CT volumetry and radiologist are not significantly different.

**Fig 4:**
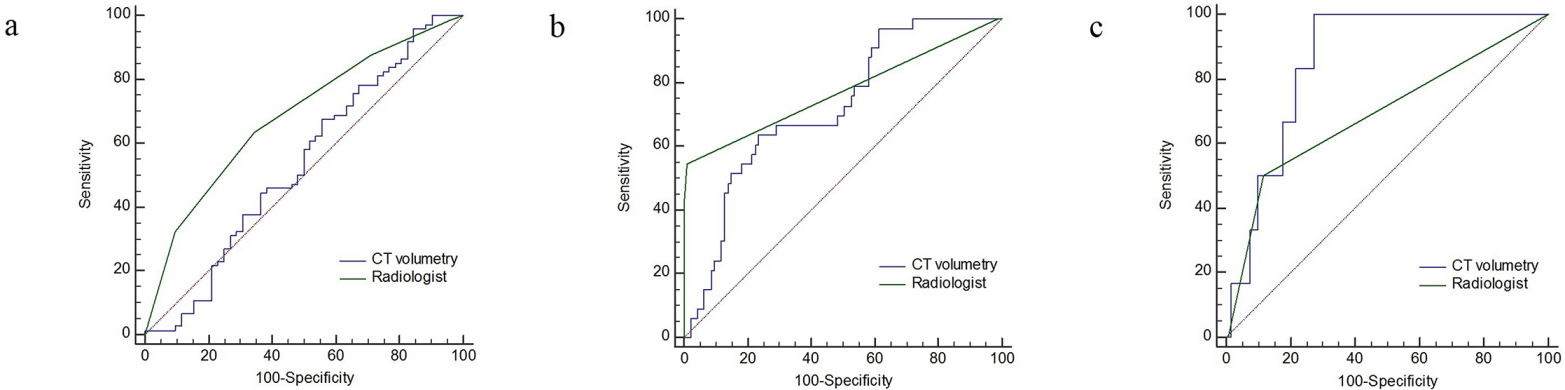
Receiver operating characteristic (ROC) curves of CT volumetry and conventional CT staging by radiologist to predict ≥ N1 (**a**), ≥ M1 (**b**), and M1b (**c**) stage of colorectal cancer. **a.** To predict ≥ N1 stage, the area under the ROC curve (Az, 0.683) of the radiologist is significantly larger than that (0.532) of CT volumetry (P = 0.015). **c.** To predict M1b stage, Az value (0.857) of CT volumetry was greater than that (0.690) of the radiologist, albeit not significant (P = 0.238).

To differentiate T staging, all Az values of Tvol were greater than those of the radiologists. In particular, the Az value of Tvol to predict pT4b stage was 0.780 and was significantly larger than that (0.591) of the radiologist (P = 0.004) (Table 4) (Fig 3). However, for N staging, Az value (0.532) of Tvol to predict ≥ pN1 stage was significantly lower than that (0.683) of the radiologist (P = 0.015) (Table 4) (Fig 4). For M staging, Az value (0.723) of Tvol to predict M1 was similar to that (0.772) of the radiologist (P = 0.495). Az value (0.857) of Tvol to predict M1b was greater than that (0.690) of the radiologist; however, statistical significance was not achieved (P = 0.238) (Table 4) (Fig 4).

**Table 4:** Comparative results of receiver operating characteristics analysis to determine TNM staging between CT volumetry and radiologist.

Stage	Differentiation	CT Volumetry			Radiologist			P Value*
		Az	95% Confidence Interval		Az	95% Confidence Interval		
			Upper	Lower		Upper	Lower	
T Stage	T1 vs ≥T2	0.958	0.907	0.986	0.891	0.823	0.939	0.514
	≤T2 vs ≥T3	0.855	0.782	0.912	0.771	0.688	0.841	0.388
	≤T3 vs ≥T4a	0.667	0.578	0.749	0.633	0.542	0.717	0.573
	≤T4a vs T4b	0.780	0.698	0.849	0.591	0.500	0.678	***0*.*004***
N Stage	N0 vs ≥N1	0.532	0.441	0.621	0.683	0.595	0.763	***0*.*015***
M Stage	M0 vs M1	0.723	0.636	0.799	0.772	0.689	0.842	0.495
	≤M1a vs M1b	0.857	0.783	0.913	0.690	0.601	0.769	0.238

TNM = tumor, node, and metastasis. Az = area under the receiver operating characteristic curve.

*P values in ***Italic Bold*** indicate a statistical significance.

### Relationship between survival and conventional TNM staging or tumor volume

**Fig 5:**
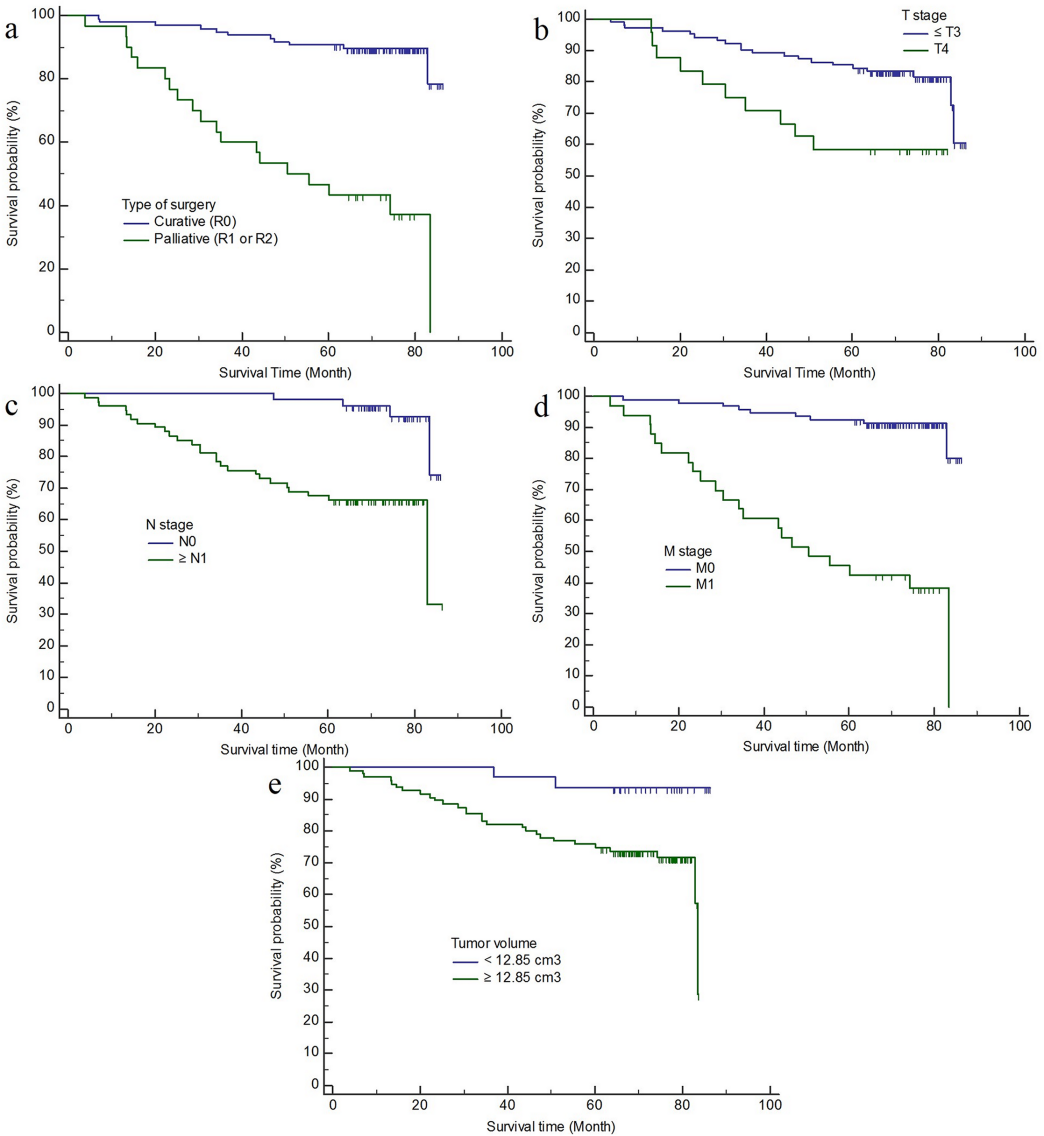
Kaplan-Meier plots for the estimated proportional survival after colorectal cancer surgery according to the type of surgery (**a**), T (**b**), N (**c**), M (**d**) staging, and tumor volume (**e**) groups. Smaller tumor volume < 12.85 cm^3^ as well as lower T (≤ T3), N (N0) and M (M0) stage were significantly associated with good prognosis after CRC surgery (P<0.05). Vertical blips on curves = censored patients.

Table 5 lists the results of Kaplan-Meier and log-rank tests for clinicopathologic and CT volumetric variables in the prognosis of 126 patients who underwent surgery for CRCs. The survival rate differed significantly with respect to tumor volume (P = 0.006) as well as tumor depth (P = 0.006), status of nodal metastasis (P<0.0001), and distant metastasis (P<0.0001), and type of surgery (P<0.0001) (Fig 5). However, no significant differences in survival rate were found with respect to age (P = 0.201) and sex (P = 0.350).

**Table 5:** Results of Kaplan-Meier and log-rank analysis of factors associated with survival after colorectal cancer surgery.

		Mean Survival ± SD (month) *	P Value*
Age at the diagnosis of CRC	< 70 (n = 91)	72.64 ± 2.52	0.201
	≥ 70 (n = 35)	74.88 ± 3.60	
Sex	Men (n = 71)	72.74 ± 2.76	0.350
	Women (n = 55)	75.48 ± 3.21	
Type of surgery	Curative resection (n = 96)	80.55 ± 1.75	***<0*.*0001***
	Palliative resection (n = 30)	52.75 ± 5.34	
pT stage	≤ T3 (n = 102)	76.61 ± 2.11	***0*.*006***
	T4 (n = 24)	60.11 ± 5.60	
pN stage	N0 (n = 52)	83.80 ± 0.98	***<0*.*0001***
	≥ N1 (n = 74)	66.11 ± 3.30	
M staging	M0 (n = 93)	81.64 ± 1.58	***<0*.*0001***
	M1 (n = 33)	52.24 ± 5.15	
Tumor volume	< 12.85 cm^3^ (n = 31)	83.63 ± 1.90	***0*.*006***
	≥ 12.85 cm^3^ (n = 95)	69.36 ± 2.53	

SD = standard deviation, CRC = colorectal cancer, pT = pathologic tumor stage, pN = pathologic node stage, M = metastasis stage.

*Median survival and P value were obtained with Kaplan-Meier method and log-rank test. P values in ***Italic Bold*** indicate statistical significance.

## Discussion

Our study results show that CTC tumor volumetry of colorectal cancer is feasible with excellent reproducibility (interclass correlation = 0.9829). The mean computational time to measure tumor volume was 4.92–6.22 minutes; subsequently, we believe that CT volumetry would not have a significant negative impact on clinical workflow. The use of CT volumetry may become more clinically viable with improvement in CT volumetry techniques such as the development of robust semi or full automated volumetry software.

In terms of T staging, tumor volume of CRC showed an incremental trend with T-stage and with significant differences between stages. In particular, tumor volume of pT4b stage was significantly larger than other stages (P<0.0001). At a cut-off value of 6.53 cm^3^, an area under the ROC curve (Az) to predict pT1 stage was 0.958 with 95.83% of sensitivity and 83.33% of specificity. Furthermore, at a cut-off value of 19.18 cm^3^, an Az value to predict pT4b stage was 0.780 and was significantly higher than (0.591) of the radiologist (P = 0.004). This result is of significant clinical impact as surgical extent should be adjusted according to a patient’s T stage. As an example, direct invasion to the adjacent organ by colorectal cancer (pT4b) may lead to an extensive en-bloc surgery that includes the invaded adjacent organ in addition to the primary tumor. Therefore, exact preoperative knowledge about the presence of adjacent organ invasion facilitates the preoperative planning for the extent of surgery which minimizes an unexpected change of surgical planning during operation.

Our study also found that tumor volumetry showed compatible performance to conventional CT staging to predict metastases as well as patients with stage IV tumors. The Az value (0.723) of tumor volumetry to predict M1 stage was similar to that (0.772) of conventional CT staging by radiologist (P = 0.495). At a cut-off value of 27.50 cm^3^, 63.64% of sensitivity and 76.34% of specificity were achieved. The Az value (0.857) of tumor volumetry to predict M1b was higher than that (0.690) of the radiologist, albeit with no statistical significance (P = 0.238). We believe that a small number of patients with M1b stage might be responsible for the insignificant statistical result. Contrary to other gastrointestinal tract cancers, the exact knowledge of the presence and number of metastatic organs is critically important for patients’ management since selective metastatectomy provides a survival benefit for patients with metastatic colorectal cancer [16]. Exact predicting M1b in which tumors metastasize to two or more organs by CT volumetry may promote radiologists to find metastatic sites using whole-body imaging such as PET/CT or whole-body MRI in a more vigorous manner.

Unexpectedly, tumor volume was not significantly different according to N stage. Larger tumors are likely to invade several layers of the colorectum due to their bulkiness and are more likely to involve the lymphatics, leading to a higher N stage. However, contrary to our expectations, tumor volumetry was found unhelpful to discriminate and predict N staging. In several previous studies for gastric cancers, compared to T and M stages showing a high accuracy, the accuracy of tumor volumetry to predict N stage was moderate [9, 11]. Furthermore, the accuracy of N staging on conventional CT is disappointing because size-based criteria has an intrinsic limitation. Normal size lymph node may harbor small metastatic foci and reactive lymph node may enlarge up to 1.5–2 cm in short axis [17, 18]. Indeed, accuracy of N staging by radiologist in our study was also low. Considering the relatively small number of study population, further studies recruiting a larger number of patients are strongly warranted to prove the exact relationship between tumor volume and nodal stage.

In our study, mean survival time was significantly longer in patients with lower T (≤ T3), N (N0), and M (M0) stage and smaller tumor burden (< 12.85 cm^3^) as well as in patients who received curative R0 resection (P<0.05). However, age and sex were not associated with patient’s survival after CRC surgery. Kikuchi et al. reported the usefulness of CT volumetry to predict patients’ survival as well as to predict TNM staging in gastric cancer [10, 11]; subsequently, CT volumetric parameter has gained acceptance as a prognostic marker for gastric cancer [9]. However, the relationship between CT volumetry and survival in patients with CRCs has not been investigated. To the best of our knowledge, this is the first report to analyze the usefulness of CT volumetry to predict patients’ survival in CRCs.

In our study, we used CT colonography for measuring tumor volume in CRCs because CT colonography is now gaining an acceptance as a non-invasive tool for the detection of CRCs as well as colonic polyps. Unlike conventional CT, CT colonography enables excellent depiction of primary tumors and also enables us to detect synchronous clinically significant (≥ 6mm) polyps or cancers. Considering that synchronous cancers are reported at 1.1–8.1% in patients with CRCs [19], the usefulness of CT colonography might be huge. Indeed, in our study, 21 patients (16.7%) had synchronous significant polyps (n = 21) and cancers (n = 3). Compared to colonoscopy, CT colonography provides thorough investigation throughout the entire colon regardless of a significant obstruction in a non-invasive manner. Indeed, colonoscopy failed to pass through the obstructing CRC in 11.9% (15/126) of our patients, not examining the proximal part of the colon.

There are several limitations in our study. First, even though CT volumetry results of our study look encouraging, it is time-consuming, subjective, and sometimes needs dedicated 3D software. However, tumor volume can now be readily and precisely assessed with the advance of MDCT technology and 3D software. In addition, an automatic or semiautomatic segmentation tool can be a potential solution to minimize the subjectiveness of a manual tracing method. Our study also demonstrated excellent reproducibility for CTC volumetry by recruiting two independent radiologists. Second, unlike in solid organ tumors, volumetric measurement in colon cancer would be affected by the degree of colonic distention. We used a standardized CTC protocol to obtain uniform distension of the colon that minimized the effect of colonic distention. Third, a prospective validation to predict patient’s survival by tumor volumetry was not performed in our study. Therefore, further prospective studies should be performed to prove our study results. Finally, in our study, only 11 patients with T1 (n = 6) or T2 (n = 5) stage were included, avoiding generalization of our study results especially to a study population with a large number of patients with low T stage.

In conclusion, CT volumetry for CRC is feasible with excellent reproducibility. CT volumetry has an acceptable accuracy to predict tumor T-stage, metastasis and patients’ survival, and may provide a useful adjunct to standard CT staging of CRCs.

## Supporting information

S1 File (XLSX)

## Abbreviations

CTC: computed tomography colonography
CRC: colorectal cancer
TNM: tumor, node, and metastasis
Tvol: Tumor volume
ROC: receiver operating characteristics
ANOVA: analysis of variance
